# Early detection of rheumatoid arthritis in rats and humans with 99mTc-3PRGD2 scintigraphy: imaging synovial neoangiogenesis

**DOI:** 10.18632/oncotarget.13953

**Published:** 2016-12-15

**Authors:** Yu Wu, Guojian Zhang, Xiangcheng Wang, Zhenfang Zhao, Tao Wang, Xuemei Wang, Xiao-Feng Li

**Affiliations:** ^1^ Department of Nuclear Medicine, Inner Mongolia Medical University Affiliated Hospital, Hohhot, Inner Mongolia, China; ^2^ Department of Nuclear Medicine, Inner Mongolia Medical University Affiliated People’s Hospital, Hohhot, Inner Mongolia, China; ^3^ PET/CT/MRI Center, The Fourth Affiliated Hospital of Harbin Medical University, Harbin, Heilongjiang, China; ^4^ Department of Radiology, University of Louisville, Louisville KY, USA

**Keywords:** rheumatoid arthritis, synovial neoangiogenesis, 99mTc-3PRGD2, 99mTc-MDP, scintigraphy

## Abstract

Objectives: To validate ^99m^Tc-labeled arginylglycylaspartic acid (^99m^Tc-3PRGD2) scintigraphy as a means to image synovial neoangiogenesis in joints afflicted by rheumatoid arthritis and to investigate its potential in the early detection and management of rheumatoid arthritis.

Methods: Rheumatoid arthritis and osteoarthritis were generated in Sprague Dawley rats by type II collagen immunization and papain injection, respectively. Rats were imaged with ^99m^Tc-3PRGD2 and ^99m^Tc- methyl diphosphonate (^99m^Tc MDP). X-ray images were also obtained and assessed by a radiologist. Immunohistochemistry of α_v_β_3_ and CD31confirmed the onset of synovial neoangiogenesis. The effect of bevacizumab on rheumatoid arthritis was followed with ^99m^Tc-3PRGD2 scintigraphy. A patient with rheumatoid arthritis and a healthy volunteer were scanned with ^99m^Tc-3PRGD2.

Results: Two weeks after immunization, a significant increase in ^99m^Tc-3PRGD2 was observed in the joints of the rheumatoid arthritis model though uptake in osteoarthritis model and untreated controls was low. ^99m^Tc-MDP whole body scans failed to distinguish early rheumatoid arthritis joints from healthy controls. The expression of α_v_β_3_ and CD31was significantly higher in the joints of rheumatoid arthritis rats compared to normal controls. In serial ^99m^Tc-3PRGD2 scintigraphy studies, ^99m^Tc-3PRGD2 uptake increased in parallel with disease progression. Bevacizumab anti-angiogenetic therapy both improved the symptoms of the rheumatoid arthritis rats and significantly decreased ^99m^Tc-3PRGD2 uptake. Significantly higher ^99m^Tc-3PRGD2 accumulation was also observed in rheumatoid arthritis joints in the patient.

Conclusions: Our findings indicate that ^99m^Tc-3PRGD2 scintigraphy could detect early rheumatoid arthritis by imaging the associated synovial neoangiogenesis, and may be useful in disease management.

## INTRODUCTION

Rheumatoid arthritis is a systemic autoimmune disease of unknown etiology which is characterized by chronic, symmetrical and erosive joint destruction. Patients with rheumatoid arthritis can develop serious joint deformities and loss of function resulting in serious disability and mortality [1]. Rheumatoid arthritis not only affects the patients’ quality of life, it also imposes a heavy financial burden on society. Currently, there are few effective therapies that can fully reverse the existing joint damage. Joint destruction can occur from 4 months to 1 year after the onset of disease, making early diagnosis and treatment vital [2]. However, imaging techniques that would allow the early detection of rheumatoid arthritis are not yet well established.

Although it is absent in other forms of arthritis, pannus formation is the most basic and important pathological feature of rheumatoid arthritis, leading to progressive cartilage destruction, bony erosion and remodeling, and hence to joint deformity and loss of function [3]. Angiogenesis is an essential feature of pannus [4], occurring as an early event in disease progression [5]. If the onset of angiogenesis can be detected, then the early diagnosis of rheumatoid arthritis may be possible, with the prospect of dramatic improvements in the clinical outcome [6].

Recent studies have shown that ligand binding to members of the integrin family of receptors plays an important role in the pathogenesis of rheumatoid arthritis [7]. In rheumatoid arthritis, α_v_β_3_ is overexpressed in synovial tissues, though it is only minimally present in the synovium of normal joints, and osteoarthritis is not associated with increased α_v_β_3_ [8]. α_v_β_3_ is highly expressed on the surface of proliferating vascular endothelial cells, neovasucular endothelial cells, osteoblasts, osteoclasts, some neutrophils and cancer cells, but is rarely expressed in existing vessels, normal tissues or dormant vascular endothelial cells [9]. α_v_β_3_ is highly expressed not only in the synoviocytes and chondrocytes of rheumatoid arthritis, but also in the osteoclasts of bone injury sites [10]. Activation of osteoclast α_v_β_3_ regulates bone reabsorption [11].

The ability of the isolated RGD tripeptide to target α_v_β_3_ has been exploited to generate an ^18^F-labeled RGD PET tracer for imaging neovascularization in breast cancer [12], and RGD has also been labeled with ^99m^Tc (^99m^Tc-3PRGD2) for SPECT imaging [13]. In this study, we generated rheumatoid arthritis in Sprague Dawley rats by type II collagen immunization. We validated ^99m^Tc-3PRGD2 imaging of synovial neoangiogenesis and we compared ^99m^Tc-3PRGD2 imaging in rheumatoid arthritis and osteoarthritis. We also explored ^99m^Tc-3PRGD2 imaging in patients with rheumatoid arthritis.

## RESULTS

**Figure 1:**
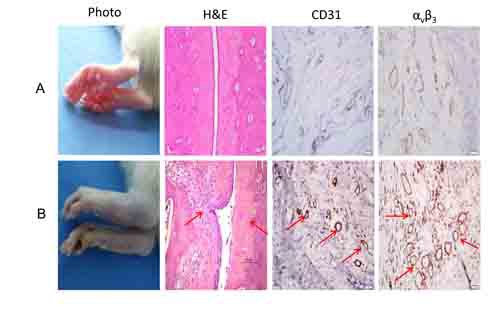
Histological changes in rheumatoid arthritis **A**. Control; **B**. Rat with rheumatoid arthritis. Sections are collected from the ankles. Regions of intense CD31 and α_v_β_3_ staining are indicated by arrows. Magnification: × 200.

One hundreds rats were injected with collagen to induce rheumatoid arthritis; of these 6 died before the observations were complete and were excluded from further analysis. Symptoms of arthritis appeared as early as 17 days after primary immunization and reached a peak by 25 days. 58 rats developed rheumatoid arthritis, a take rate of 62% (58/94), in agreement with a previous report [20]. Figure 1 shows swelling of the ankles and toes in a treated rat 15 days after primary immunization. H&E staining revealed narrowing of the joint cavity, with elevated CD31 and α_v_β_3_ expression in synovial tissue compared to the control. Weight gain following immunization was significantly delayed compared to control rats (data not shown).

**Figure 2:**
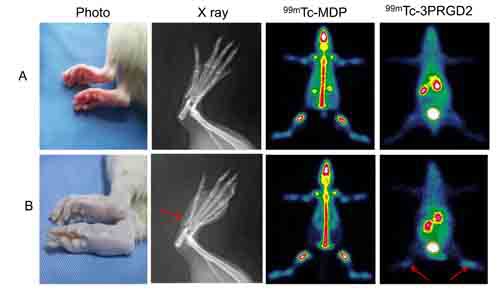
Receptor imaging with ^99m^Tc-3PRGD2 **A**. Control; **B**. Rat with rheumatoid arthritis. X ray and ^99m^Tc-MDP bone scans are compared with ^99m^Tc-3PRGD2 imaging. An abnormal increase in ^99m^Tc-3PRGD2 uptake is evident in rheumatoid arthritis, as indicated by the arrow.

**Figure 3:**
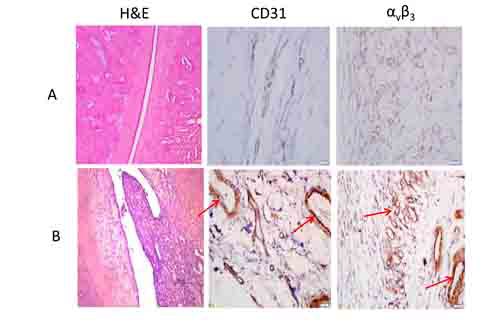
Histological changes matched to ^99m^Tc-3PRGD2 imaging **A**. Control; **B**. Rat with rheumatoid arthritis. Images are obtained from the animals scanned in Figure 2. Regions of intense CD31 and α_v_β_3_ staining are indicated by arrows. Magnification: × 200.

^99m^Tc-3PRGD2 and ^99m^Tc-MDP were compared as imaging agents in 5 diseased and 5 control rats. As shown in Figure 2 and Supplemental Figure 1, significantly higher ^99m^Tc-3PRGD2 uptake was observed in the rheumatoid (0.57±0.18) compared to the disease-free joints (0.21±0.06, *P* < 0.01); uptake is quantified as the ratio of activity in the joint relative to the mediastinum. However, ^99m^Tc-MDP imaging could not distinguish between diseased and healthy joints. Immunohistochemical staining revealed increased CD31 and α_v_β_3_ in diseased joints compared to controls (Figure 3). H&E staining revealed heavy infiltration of inflammatory cells in the lesion synovium and cartilage damage in the rheumatoid arthritic joint (Figure 3). We also obtained ^99m^Tc-3PRGD2 images of rats with osteoarthritis. ^99m^Tc-3PRGD2 accumulation in osteoarthritic joints (0.24±0.05) was not significantly different from that in normal joints (Suppl. Figure 2), and α_v_β_3_ and CD31 staining was similar to untreated rats (Suppl. Figure 2).

**Figure 4:**
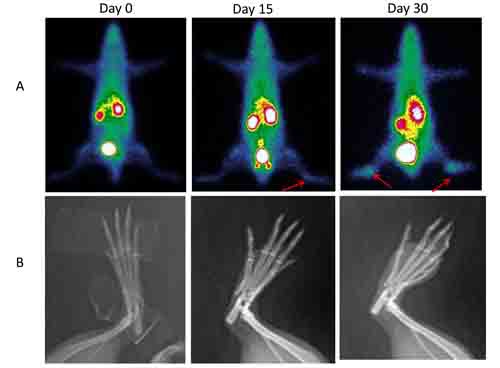
Temporal changes in rheumatoid arthritis monitored by serial ^99m^Tc-3PRGD2 and X-ray imaging **A**. ^99m^Tc-3PRGD2 scintigraphy prior to, and 15 and 30 days after immunization Arrow indicates increased joint uptake. **B**. Matched X-ray images.

Serial ^99m^Tc-3PRGD2 SPECT scans were conducted in 20 collagen-immunized rats, of which 13 developed pathologically-confirmed rheumatoid arthritis and were included in the report. Images were obtained before, 15 days and 30 days after immunization. A representative SPECT image is shown in Figure 4, along with the matched X-ray. Tracer uptake in the joints increased with time, being 0.41 ± 0.23 on day 15 and 0.51 ± 0.18 on day 30, significantly higher than pre-immunization status (0.22±0.19, *P* < 0.01). Separate sets of imaging were also presented to support the findings (Suppl. Figure 3-6)

**Figure 5:**
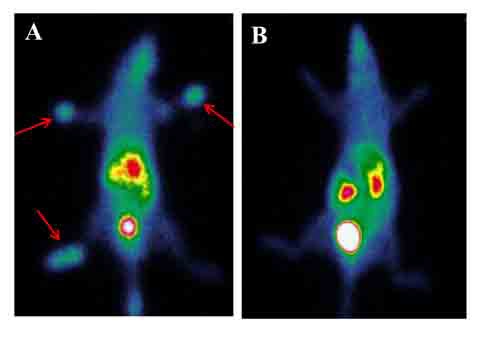
Bevacizumab reduces uptake of ^99m^Tc-3PRGD2 in rheumatoid arthritis **A**. ^99m^Tc-3PRGD2 imaging of a rat with established rheumatoid arthritis prior to bevacizumab treatment; **B**. ^99m^Tc-3PRGD2 imaging 2 weeks after bevacizumab administration.

A group of 40 rheumatoid arthritis rats received bevacizumab therapy: as shown in Figure 5, this resulted in a dramatic reduction of ^99m^Tc-3PRGD2 uptake in the joints. Overall ^99m^Tc-3PRGD2 joint activity decreased from 0.53 ± 0.22 to 0.22 ± 0.02 (*P* < 0.01) accompanied with a significant improvement in symptoms. A full report on bevacizumab as a therapeutic option will be presented elsewhere.

**Figure 6:**
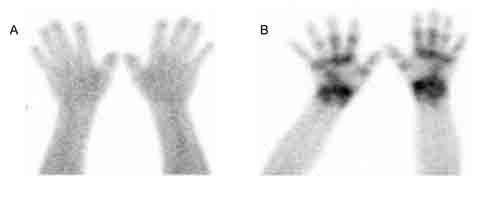
^99m^Tc-3PRGD2 scintigraphy in a patient with rheumatoid arthritis and a healthy volunteer ^99m^Tc-3PRGD2 scintigraphy of the forearms and hands of a healthy volunteer **A**. and a 54-yr-old female with active rheumatoid arthritis **B**.

^99m^Tc-3PRGD2 imaging was also performed in a female patient with rheumatoid arthritis and a healthy volunteer. Abnormally high ^99m^Tc-3PRGD2 accumulation was observed in multiple hand joints of the patient compared to the volunteer (Figure 6).

## DISCUSSION

Receptor imaging combines the high specificity of ligand-receptor binding with the high sensitivity of radioisotope detection. This study takes advantage of the specific affinity of ^99m^Tc-3PRGD_2_ for integrin α_v_β_3_ and provides a convenient new imaging method for the early diagnosis of rheumatoid arthritis and disease management. We found that in rats with rheumatoid arthritis, joint tissue showed increased expression of CD 31 and α_v_β_3_, indicating increased angiogenesis as an early feature of the disease (Figure 1). Consistent with this, ^99m^Tc-3PRGD_2_ uptake was significantly increased in rheumatoid arthritis compared to control rats and animals with osteoarthritis (Figure 2, Figure 3, Suppl.Figure 1, Suppl.Figure 2). Therefore, ^99m^Tc-3PRGD_2_ is a promising agent for the early diagnosis of rheumatoid arthritis. Additionally, ^99m^Tc-3PRGD_2_ was able to report on the success of antiangiogensis therapy of rheumatoid arthritis (Figure 5).

Rheumatoid arthritis is a common chronic inflammatory disease characterized by damage of bones and joints. The infiltration of inflammatory cells, pannus formation and angiogenesis are the early specific pathological changes [21]. We used type II collagen immunization of Sprague Dawley rats to establish a model of rheumatoid arthritis. The clinical manifestation, pathological and immunological changes found in this model are similar to those in humans [22–24].

Of note, only 62% rats developed rheumatoid arthritis after type II collagen immunization. Of the 20 rats assigned to the serial imaging study, 7 did not develop disease over the time of the experiment, and in these animals ^99m^Tc-3PRGD2 uptake was not significantly different from the controls (data not shown).

In the early stage of rheumatoid arthritis in rats, there is no bone destruction, which limited the power of X-ray imaging for early disease detection. ^99m^Tc-MDP bone scan has been frequently used for bone and joint diseases, but our findings indicate that ^99m^Tc-MDP may not be useful for rheumatoid arthritis (Figure 2). ^99m^Tc-3PRGD2 scintigraphy was clearly superior to other convenient imaging modalities, and its success in imaging arthritis in a patient is a further positive result.

## CONCLUSIONS

^99m^Tc-3PRGD2 is an imaging agent that enables the early detection of synovial neoangiogenesis in an experimental model of rheumatoid arthritis and hence may be useful in the clinic for managing treatment of the disease.

## MATERIALS AND METHODS

### Rat models of rheumatoid arthritis and osteoarthritis

All experiments were performed using 8-week-old female Sprague Dawley rats (body weight 180~200g, purchased from Beijing Vital River Laboratory Animal Technology). A total of 140 rats were purchased, maintained and used according to institutional guidelines. The experimental protocol was approved by the Institutional Animal Care and Use Committee of Inner Mongolia Medical University. Animals were housed 2 per cage and kept in the institutional animal facility at a constant temperature and humidity. Food pellets and water were provided *ad libitum*.

Rheumatoid arthritis was generated by type II collagen (Sigma) immunization as described previously *(14)*. Briefly, type II collagen was dissolved in 0.01mol/L acetic acid overnight at 4°C. Collagen was then emulsified by complete Freund adjuvant (Sigma) and intradermally injected at the tail tip (0.2ml, 1mg/ml) for primary immunization. Two weeks later, type II collagen solution emulsified by incomplete Freund adjuvant (Sigma) was intradermally injected to the inguinal region at the same dose. A total of 100 rats were immunized to generate rheumatoid arthritis model.

Osteoarthritis was established by intra-knee joint cavity injection of L-cysteine papain (20 µl) in 20 rats [15]. 20 control animals received two intradermal injections of 0.9% saline.

### Observation of general conditions of rats

Clinical symptoms including general condition, mobility, hair color change and joint swelling were observed and recorded every day. Body weights were recorded every 5 days. The degree of whole-body joint lesions and the arthritis index score were recorded every 5 days. Arthritis index scores were established according to previously described criteria [16]: 0, no swelling; 1, slight swelling of the toe joint; 2, toe joint swelling and toe swelling; 3, feet swelling below the ankle joint; and 4, feet and ankle joint swelling. In this study, the standard of success model was defined as total arthritis index scores greater than 3 after 30 days post-immunization.

### ^99m^Tc-3PRGD2 and ^99m^Tc-Methyl diphosphonate imaging

3PRGD2 (Isotopic Laboratory Peking University) was labeled with ^99m^TcO4^-^ eluted from an in-house ^99^Molybdenum-^99m^Technetium generator (China Institute of Atomic Energy) as previous described [17]. Briefly, ^99m^TcO4^-^ solution was added to a vial containing 3PRGD2 solids with frequent vigorous shaking for complete dissolution. The vial was incubated in a water bath at 100°C for 20 minutes and then cooled at room temperature. The radiochemical purity of ^99m^Tc-3PRGD2 was always greater than 95%.

^99m^Tc-3PRGD2 scintigraphy was performed one day before and 15 and 30 days after the first type II collagen immunization. Scintigraphy was accomplished with a dual-head detector SPECT/CT (Millennium VG, Hawkeye; GE Healthcare) equipped with low-energy and high-resolution collimators (peak energy 140 keV, window width 20%). Approximately 1 hour after ^99m^Tc-3PRGD2 (11.1MBq/kg) injection via the caudal vein, animals were anesthetized by inhalation of 1.5 % isoflurane-air mixture. A 6 minute planar scan was conducted and SPECT data was acquired into a 128 × 128 matrix. ^99m^Tc-3PRGD2 images of rheumatoid arthritis, osteoarthritic and disease-free rats were acquired. ^99m^Tc-Methyl diphosphonate (MDP) bone scans were acquired in the same rheumatoid arthritic rats 2 days later. ^99m^Tc-MDP (11.1MBq/kg) was imaged using the same protocol as ^99m^Tc-3PRGD2 imaging.

^99m^Tc-3PRGD2 whole body scans were performed in a heathy volunteer and a patient with rheumatoid arthritis. The human studies were approved by the Institutional Review Board of Inner Mongolia Medical University and the local ethics committee. Written consent was obtained from the patient and the volunteer.

For studies where ^99m^Tc-3PRGD2 imaging was used to follow the effect of bevacizumab on rheumatoid arthritis, bevacizumab (30 mg/kg) was intravenously injected weekly for two weeks. Control animals received intravenous saline on the same schedule. ^99m^Tc-3PRGD2 imaging was performed pre- and two weeks after initial therapy following the protocol described above.

The images were processed using a post-processing system workstation. Tracer uptake in the joints was expressed semi-quantitatively as the ratio of activity in the limb joints to the mediastinum. Images were read by two board certified nuclear medicine physicians who were blinded to the pathologic findings. In case of disagreement, a third nuclear medicine physician made a diagnostic conclusion.

### X ray imaging

Rats were given an X-ray exam immediately after each SPECT scan while still under anesthesia. Images were interpreted by board certified radiologists who were familiar with rat anatomy and blinded to the final pathologic findings. The assessment included peri-articular soft tissue swelling, articular surface smoothness and joint space narrowness.

### Histological analysis of joint tissues

Animals were sacrificed by carbon dioxide. The knee, ankle and paw joints were harvested and fixed in 10% phosphate-buffered formalin for 48 hours. Bones were decalcified in 8% EDTA solution. Fixed and decalcified specimens were processed and 4 µm paraffin sections were cut with a Leica RM2135 microtome (Leica) for staining with hematoxylin, eosin and toluidine blue. Scoring of synovium pathological appearance was done by two skilled pathologists who observed the synovial hyperplasia, inflammatory cell infiltration, pannus formation and damage to bone or cartilage. CD31 and α_v_β_3_ immunohistochemical test kits (Beijing Bioss Biotechnology Co.) were used according to the manufacturer's instructions [18, 19].

### Statistical analysis

^99m^Tc-3PRGD2 uptake was expressed as mean ± standard deviation. Statistical significance was examined by a 2-tailed Student's *t* test. A *p* value of less than 0.05 was considered as statistically significant.

## SUPPLEMENTARY MATERIALS FIGURES AND TABLES


