# Supplementary material for: Early detection of rheumatoid arthritis in rats and humans with 99mTc-3PRGD2 scintigraphy: imaging synovial neoangiogenesis
Source: Oncotarget. 2016 Dec 15;8(4):5753–60. doi: 10.18632/oncotarget.13953 (PMC5351586; doi:10.18632/oncotarget.13953)
Supplement: Supplementary file 1 [file oncotarget-08-5753-s001.pdf]

Early detection of rheumatoid arthritis in rats and humans with ^{99m}Tc-3PRGD2 scintigraphy: imaging synovial neoangiogenesis

Supplementary Material

Supplemental Figure 1

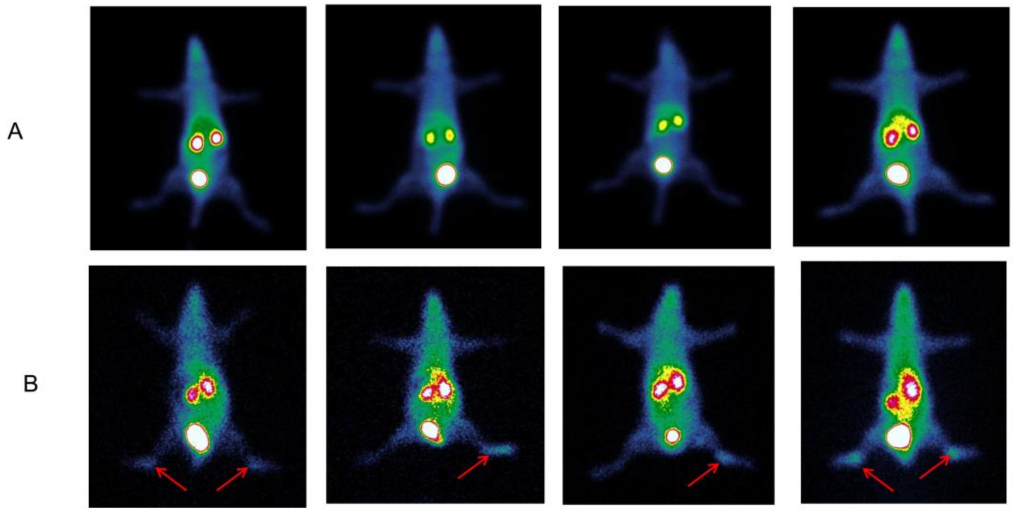

Supplemental Figure 1. Receptor imaging with ^{99m}Tc-3PRGD2 in 4 health rats as controls and 4 rats with rheumatoid arthritis. A: Control; B: Rat with rheumatoid arthritis. An abnormal increase in ^{99m}Tc-3PRGD2 uptake is evident in rheumatoid arthritis as indicated by the arrows.

Supplemental Figure 2

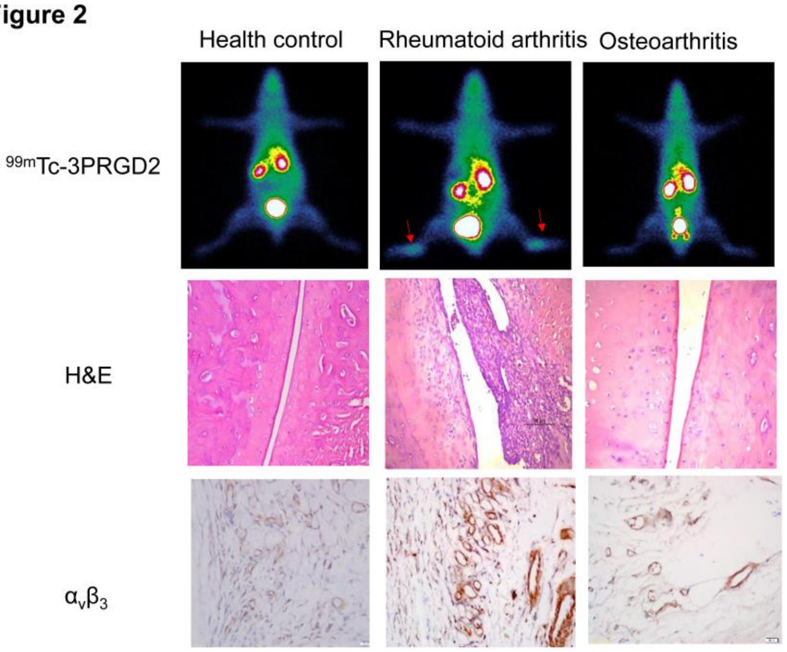

Supplemental Figure 2. Comparison ^{99m}Tc-3PRGD2 uptake among rheumatoid arthritis, osteoarthritis and health rats relating to histological findings. Increase in ^{99m}Tc-3PRGD2 uptake is observed in rheumatoid arthritis joints, ^{99m}Tc-3PRGD2 accumulation osteoarthritis joint was not significant different from that in normal joint; overexpression of $\alpha_v\beta_3$ was found only in rheumatoid arthritis joints.

Supplemental Figure 3

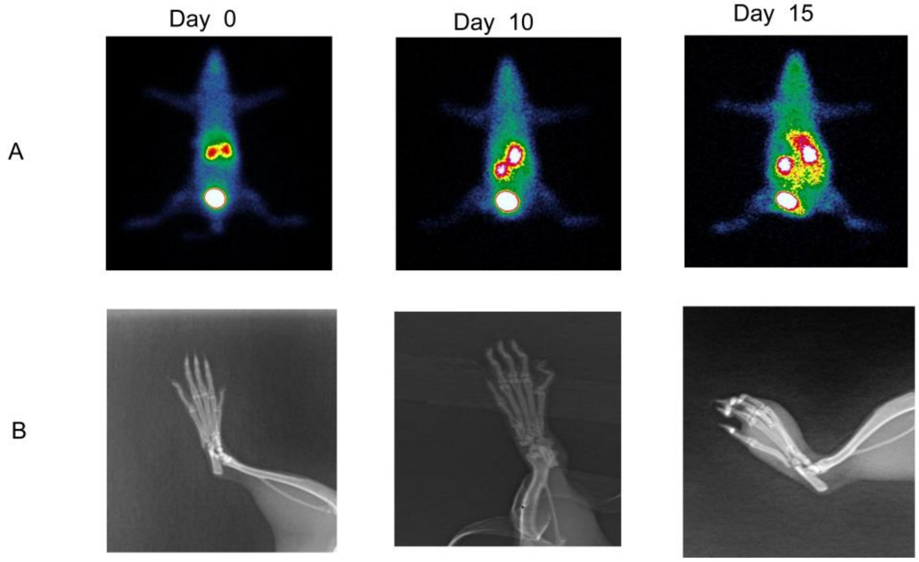

Supplemental Figure 4

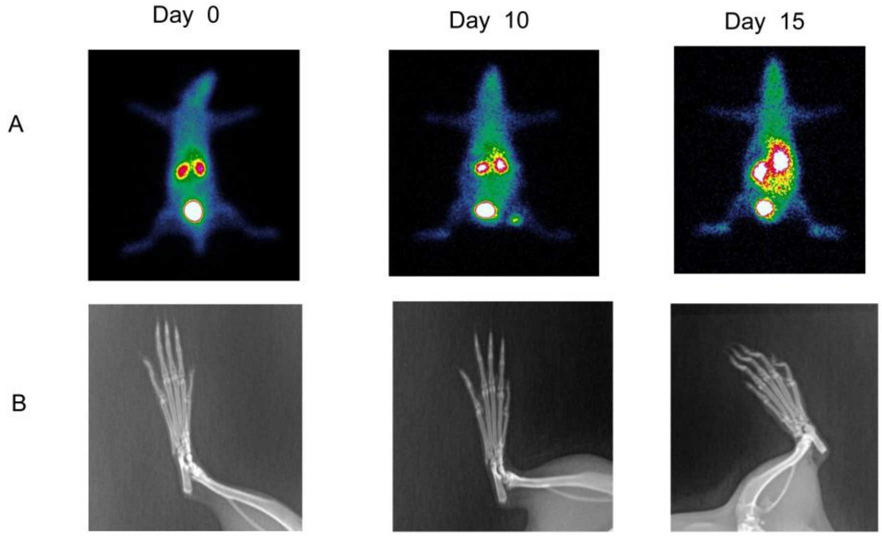

Supplemental Figure 5

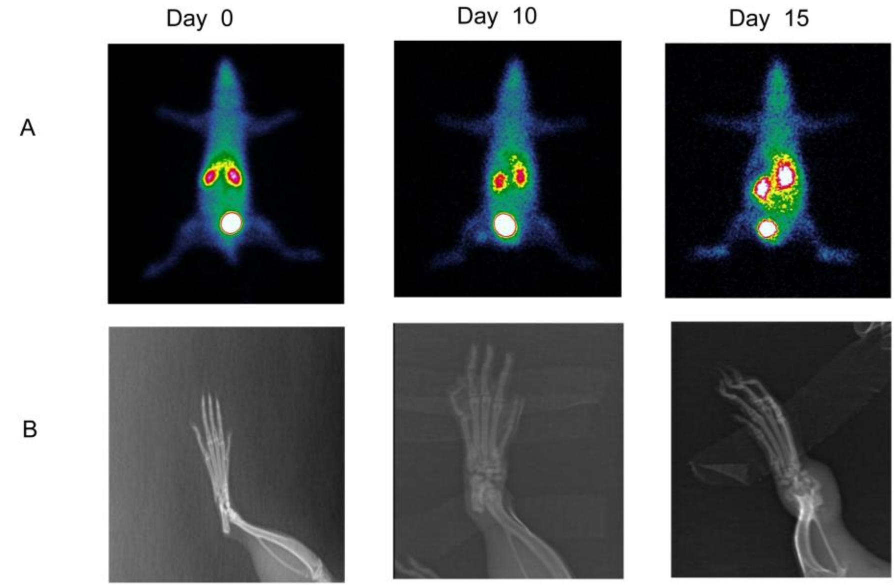

Supplemental Figure 6

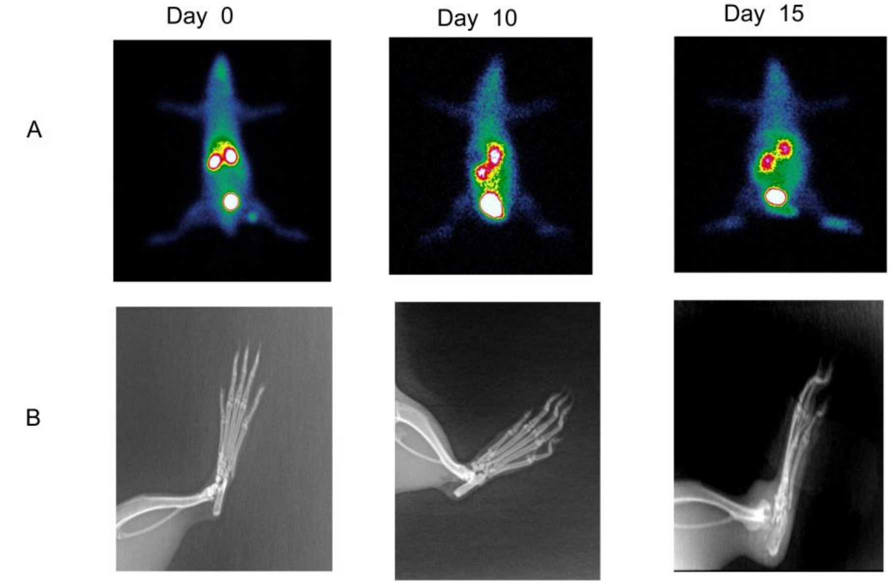

Supplemental Figure 3-6.

Temporal changes in rheumatoid arthritis monitored by serial ^{99m}Tc-3PRGD2 and X-ray imaging in 4 rats in addition to the data presented in Figure 4.

A: ^{99m}Tc-3PRGD2 scintigraphy prior to, and 15 and 30 days after immunization. Arrow indicates

increased joint uptake. B: Corresponding X-ray images.
